# Cross-modal semantic autoencoder with embedding consensus

**DOI:** 10.1038/s41598-021-92750-7

**Published:** 2021-10-13

**Authors:** Shengzi Sun, Binghui Guo, Zhilong Mi, Zhiming Zheng

**Affiliations:** 1 Beijing Advanced Innovation Center for Big Data and Brain Computing and NLSDE, Beihang University, Beijing, 100191 China; 2 Peng Cheng Laboratory, Shenzhen, 518055 Guangdong Province China; 3 LMIB and School of Mathematical Sciences, Beihang University, Beijing, 100191 China

**Keywords:** Energy science and technology, Mathematics and computing

## Abstract

Cross-modal retrieval has become a topic of popularity, since multi-data is heterogeneous and the similarities between different forms of information are worthy of attention. Traditional single-modal methods reconstruct the original information and lack of considering the semantic similarity between different data. In this work, a cross-modal semantic autoencoder with embedding consensus (CSAEC) is proposed, mapping the original data to a low-dimensional shared space to retain semantic information. Considering the similarity between the modalities, an automatic encoder is utilized to associate the feature projection to the semantic code vector. In addition, regularization and sparse constraints are applied to low-dimensional matrices to balance reconstruction errors. The high dimensional data is transformed into semantic code vector. Different models are constrained by parameters to achieve denoising. The experiments on four multi-modal data sets show that the query results are improved and effective cross-modal retrieval is achieved. Further, CSAEC can also be applied to fields related to computer and network such as deep and subspace learning. The model breaks through the obstacles in traditional methods, using deep learning methods innovatively to convert multi-modal data into abstract expression, which can get better accuracy and achieve better results in recognition.

## Introduction

With the development of Internet technology, a large amount of multi-media data is constantly emerging, which brings great challenges to information retrieval. Data sources include texts, images, videos and audios, etc^1^. In fact, image and text information is quite common. Traditional single-modal retrieval cannot solve the compatibility problem, since it only returns the original data for query in the same way and cannot meet the retrieval needs. Therefore, cross-modal information retrieval has become a topic of popularity, and methods have emerged and developed rapidly with the goal of effectively retrieving different information patterns, such as retrieving parts of images with texts^2,3^.

Cross-modal retrieval utilizes various types of data to query different forms of information. To perform cross-modal retrieval, the key issue is to consider the semantic similarity between different forms of data. The heterogeneity between different modalities becomes a great challenge. Currently, existing image-text cross-modal retrieval methods include paired models^4^, sorting^5,6^, mapping^7,8^, and graph embeddings^9,10^. Besides, probabilistic models, metric learning methods, and subspace learning methods are applied to many data sets. Probabilistic methods learn multi-modal correlation by modeling joint multimodal data distributions^11^. The metric learning method learns to calculate the distance metric between different modalities^12^. Some classic methods^2,13^ project data into a public space. To obtain good retrieval results, embedding methods are used to retain both semantic and original feature information^14^. Original feature information^15,16^ supplements semantic information by providing other internal modal relationships. Zhou et al.^17^ proposed a potential semantic sparse hashing method(LSSH), which combines sparse coding and matrix decomposition to obtain a potential shared semantic space. In deep methods, convolutional neural network(CNN) are commonly used to generate images. The semantic part embeds features for each word and generates text through text CNN or recurrent neural network(RNN)^18^.

The lack of semantic information will lead to limited retrieval results. Partial regression methods such as SCM^14^, LCFS^7^, and LGCFL^19^ focus on preserving semantic information. However, the above three methods can only be used to deal with single-modal situations, ignoring the correlation between tags in multi-modal information. In addition, fixing the public space as a label space will decrease the efficiency when increasing amount of data.

To solve the above problems and achieve an efficient information retrieval, we propose a learning method called cross-modal semantic autoencoder with embedding consensus (CSAEC). First, the paired image and text data are embedded and mapped into a unified space, called mapping consensus, while retaining the original feature information and semantic information. Further, through feature extraction, the data is converted into corresponding semantic code vectors. To remove redundant information, the multi-label space of high-dimensional data is compressed, and parameters are introduced to achieve denoising. Then, feature projections are learned using paired encoder–decoders, one for image form and the other for text form. The similarity between the projected information is associated with the semantic code vector. Further, the objective function is minimized, and the matrix is subjected to regularization and sparse constraints to balance the reconstruction error.

## Results

We show the performance of the proposed method with experimental results. On four multi-modal data sets, CSAEC is compared with other existing methods to verify its effectiveness. Specific results are analyzed through various index values.

### Datasets and compared methods

*WIKI*^14^ The total number of entries in each version of Wikipedia has exceeded 53 million, supporting various languages, of which Chinese Wikipedia has more than 1.13 million entries. We choose 2200 image-text pairs for training and 700 image-text pairs for testing.

*TVGraz*^20^ It contains 2594 image-text pairs. We choose more than 10 words and select 2500 image-text pairs. We set 4000-dimensional feature for images and 8300 vertices for texts.

*NUS-WIDE*^21^ We set 60,000 image-text pairs for training and 10000 image-text pairs for testing. For the texts data, we choose 1000-dim tag occurrence features.

*MIRFLICKR*^22^ It contains 25000 instances for images and textual tags. We set 38 classes for image-text data and use the train-test split. By feature selection, 3000-dim tag frequency features are used for text representations.

Using four multi-modal data sets WIKI^14^, TVGraz^20^, NUS-WIDE^21^, and MIRFLICKR^22^, we compare CSAEC with five existing methods, CCA^23^, BLM^2^, LCFS^7^, LGCFL^19^, JFSSL^10^. CCA and BLM are two unsupervised models that use paired information to maximize the correlation between projection vectors. LCFS, LGCFL, and JFSSL are three supervised models that use semantic class information to directly associate data from one modality with data from another modality. The LGCFL method can learn the discriminant by moving the label space to increase the distance between classes and adding sparse constraints on the group during the regression process^19^. The JFSSL method adds a regular term to the projection space^10^.

### Parameter settings

The spatial dimensions of WIKI, TVGraz, NUS-WIDE, and MIRFLICKR are set to 10, 20, 10, and 40, respectively. We constantly adjust parameters within the range of 0.001, 0.01, 0.1, 1, 10 to analyze the performance of CSAEC. For several other methods, we set the parameter values according to the corresponding data set. For the data set, we randomly divide it into parts, one of which is the test data, and the rest is the unlabeled pool for active selection. The random data partition is repeated for ten times and average results over them are reported as the final model evaluation.

### Complexity analysis

We set $n \ge d$. The complexity of eigenvalue decomposition is $O(n^3)$. When *n* is large, we can get the results with iterative algorithms to prove the precision of our proposed methods. The largest *d* eigenvalues may exit with different datasets. Obviously, the size of feature dimension influence the complexity. We just caculate with $O(knd^3)$ and *k* is the number of iterations.

### Mean average precision (MAP) results of different methods

Mean Average Precision (MAP) is used to evaluate the validity of the retrieval results of different methods. *R* is the threshold for Precision–Recall (PR) Curves. Assuming that there are some positive examples in the datasets, we can get the corresponding values *r*. For each value of *r*, we can calculate the maximum precision when $r>R$. In order to verify the performance of CSAEC, two types of directional cross-pattern retrieval tasks were performed: image-text query and text-image query. If the labels of the two types of data points are the same, the information is considered to have the relevance.

The methods are compared on the WIKI dataset. It can be observed from Table 1 that the performance of the CSAEC method in this paper has improved significantly.

**Table 1:** MAP results of different methods (WIKI).

$\hbox {R}=40$	Image-text	Text-image	Average	Average rank	$\hbox {R}=\hbox {all}$	Image-text	Text-image	Average	Average rank
CCA	0.436	0.545	0.492	4.375	CCA	0.428	0.417	0.424	4.75
BLM	0.443	0.532	0.495	4.75	BLM	0.445	0.438	0.443	4.375
LCFS	0.461	0.564	0.517	2.75	LCFS	0.459	0.441	0.442	2.375
LGCFL	0.473	0.569	0.526	4.375	LGCFL	0.468	0.453	0.461	4.75
JFSSL	0.474	0.572	0.526	3.75	JFSSL	0.462	0.457	0.468	3.75
CSAEC	0.489	0.582	0.534	1	CSAEC	0.478	0.481	0.479	1

Average ranks by each algorithm provide avaluable comparison.Let $r_{i(m)}^j$ denotes that the rank of *j*th of *m* algorithm applied to the ith dataset. Then the average rank of *k* algorithm can be expressed as $R_m^j = {\textstyle {1 \over n}}\sum \nolimits _i {r_{i(m)}^j}$ . Then establish a null assuming that all algorithms have strong similarities, which states that the ranks $R^j$ should be equaled. The Friedman test testifies whether the calculated average ranks should have significantly difference from the mean rank expected under the null hypothesis.

When $\hbox {R}=40$, the Friedman statistic can be calculated as

$$\begin{aligned} \chi _F^2= & {} \frac{{12n}}{{k(k + 1)}} \left[ {\sum \limits _j {R_j^2 - \frac{{k{{(k + 1)}^2}}}{4}} } \right] \\= & {} \frac{{12 \cdot 4}}{{6 \cdot 7}} \left[ {4.375^2} + {4.75^2} + {2.75^2} + {4.375^2} + {3.75^2} + 1 - \frac{{6 \cdot {7^2}}}{4}\right] = 11.392 \end{aligned}$$

Imam and Davenport can have a better statistic value than Friedman statistic which generates a conservative behavior^24^

$$\begin{aligned} {F_{F_1}} = \frac{{(n - 1)\chi _{F_1}^2}}{{n(k - 1)-\chi _{F_1}^2}} =\frac{{3 \cdot 11.392}}{{4 \cdot 6 - 11.392}} = 2.71 \end{aligned}$$

When $\hbox {R}=\text{all}$, the Friedman statistic can be calculated as

$$\begin{aligned} \chi _{F_2}^2= & {} \frac{{12n}}{{k(k + 1)}}\left[ {\sum \limits _j {R_j^2 - \frac{{k{{(k + 1)}^2}}}{4}} } \right] \\= & {} \frac{{12 \cdot 4}}{{6 \cdot 7}} \left[ {4.75^2} + {4.375^2} + {2.375^2} + {4.75^2} + {3.75^2} + 1 - \frac{{6 \cdot {7^2}}}{4}\right] = 11.392 \\ {F_{F_2}}= & {} \frac{{(n - 1)\chi _{F_2}^2}}{{n(k - 1)-\chi _{F_2}^2}} =\frac{{3 \cdot 11.392}}{{4 \cdot 6 - 11.392}} = 2.71 \end{aligned}$$

With four data sets and six algorithms, $F_F$ is distributed according to the *F*-distribution with $(6-1)=5$ and (6 − 1) $(4-1)=15$ degrees of freedom. The *p*-value calculated with *F*(5, 15) distribution has proved the null hypothesis can be rejected at a high level of significance. The reason may be that CSAEC uses the embedding matrix while preserving the original features and semantic information. Semantic information provides interactive information between modalities and information within each modality, while original feature information takes into account of the similarity between modalities.

As can be seen from Table 2, on TVGraz dataset, CSAEC also achieved the best results for the two types of retrieval tasks. Our method improves the performance of image query text tasks better than text query images. Compared with other methods, the query results are improved.

**Table 2:** MAP results of different methods (TVGraz).

$\hbox {R}=40$	Image-text	Text-image	Average	$\hbox {R}=\hbox {all}$	Image-text	Text-image	Average
CCA	0.629	0.624	0.627	CCA	0.612	0.603	0.619
BLM	0.637	0.625	0.634	BLM	0.623	0.618	0.626
LCFS	0.647	0.647	0.651	LCFS	0.637	0.625	0.634
LGCFL	0.658	0.641	0.653	LGCFL	0.649	0.636	0.641
JFSSL	0.654	0.645	0.656	JFSSL	0.654	0.649	0.657
CSAEC	0.672	0.653	0.671	CSAEC	0.663	0.659	0.674

Table 3 shows the MAP of each method on the NUS-WIDE dataset. The LGCFL and CSAEC methods perform better than CCA because both consider semantic information. The NUS-WIDE dataset is larger than the WIKI and MIRFLICKR datasets, so the semantic information has more interaction in NUS-WIDE, and similar information between different modal data can be found as much as possible.

**Table 3:** MAP results of different methods (NUS-WIDE).

$\hbox {R}=40$	Image-text	Text-image	Average	$\hbox {R}=\hbox {all}$	Image-text	Text-image	Average
CCA	0.782	0.775	0.768	CCA	0.759	0.762	0.764
BLM	0.859	0.836	0.849	BLM	0.835	0.842	0.833
LCFS	0.843	0.828	0.837	LCFS	0.834	0.833	0.835
LGCFL	0.782	0.778	0.791	LGCFL	0.774	0.776	0.772
JFSSL	0.767	0.769	0.765	JFSSL	0.753	0.767	0.754
CSAEC	0.867	0.861	0.863	CSAEC	0.859	0.852	0.848

On the MIRFLICKR dataset, it can be seen from Table 4 that the MAP value of this method is better than other methods, and the effect of JFSSL is second. The CSAEC method has the ability to retain both original features and semantic information, and learns the feature code vector of the semantic tag space. This shows that CSAEC and JFSSL are effective for querying spatial information with labels.

**Table 4:** MAP results of different methods (MIRFLICKR).

$\hbox {R}=40$	Image-text	Text-image	Average	$\hbox {R}=\hbox {all}$	Image-text	Text-image	Average
CCA	0.873	0.856	0.859	CCA	0.732	0.739	0.734
BLM	0.861	0.858	0.864	BLM	0.742	0.746	0.751
LCFS	0.895	0.873	0.891	LCFS	0.756	0.754	0.757
LGCFL	0.895	0.889	0.891	LGCFL	0.768	0.765	0.762
JFSSL	0.904	0.881	0.893	JFSSL	0.778	0.782	0.784
CSAEC	0.925	0.981	0.956	CSAEC	0.798	0.824	0.815

### Precision–Recall (PR) curves of different methods

**Figure 1:**
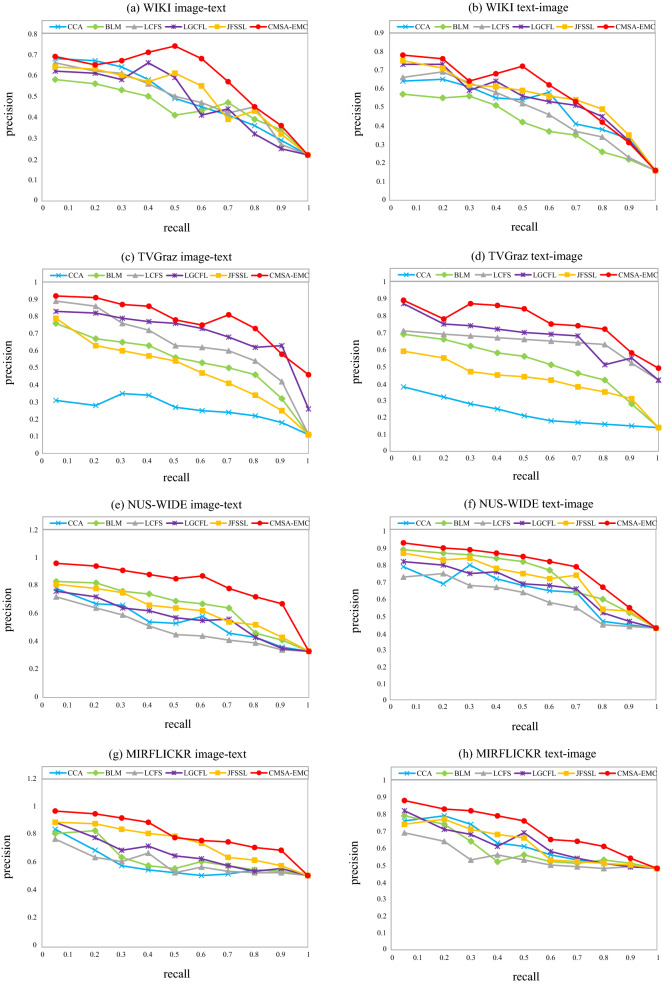
Precision–Recall (PR) curves of different methods. On the four datasets, We compared CSAEC with other five methods to prove the better results. The method completes the query task and improves the retrieval performance.

It can be seen from Fig. 1 that for the image-text query task, the overall CSAEC query effect exceeds almost all other methods. On the MIRFLICKR dataset, the minimum accuracy of each method is higher. On the NUS-WIDE dataset, the performance advantage of CSAEC is more obvious. Overall, CSAEC improves the performance of image query text tasks. For text-image query tasks, CSAEC has higher recall rate than the other methods on the four benchmark data sets.

### Parameter sensitivity

**Figure 2:**
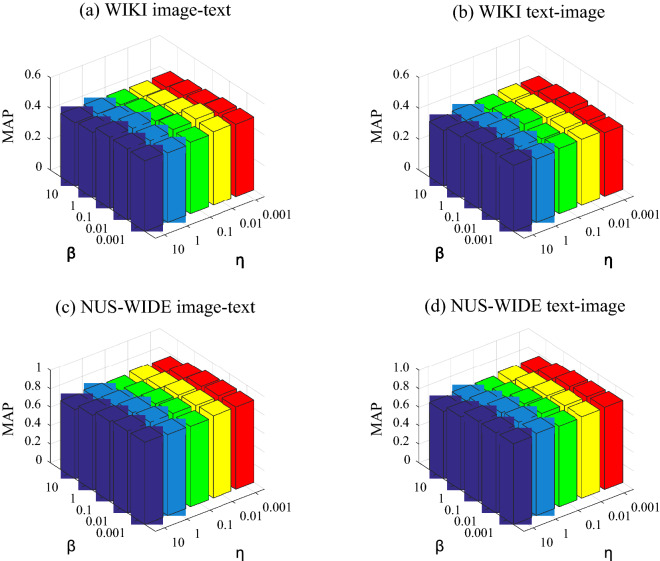
Parameter sensitivity. The two parameter values are adjusted within the range of 0.001, 0.01, 0.1, 1, 10. The parameters’ query performance is relatively sensitive.

**Figure 3:**
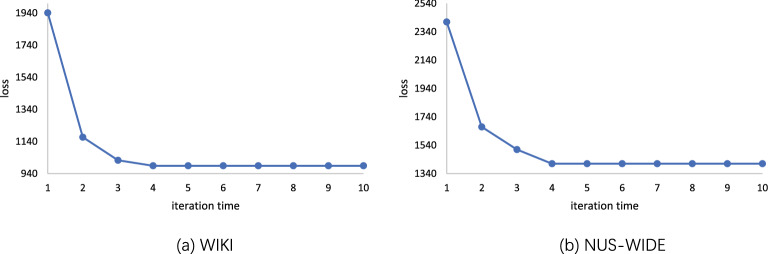
Convergence curves. We plot the convergence curves of iterative algorithm with respect to the loss value. With very few iterations, the losses become small and stable.

In Fig. 2, we analyze the impact of parameters. On the WIKI and NUS-WIDE datasets, the two parameter values are adjusted within the range of 0.001, 0.01, 0.1, 1, 10, and their changes are shown in Fig. 3. It can be seen that when the parameters change, the effect of CSAEC will be different, and its query performance is more sensitive than other methods. When the range is from 0.001 to 1, this method can get better results.

### Loss analysis

Figure 3 shows the convergence loss curve of the method in this paper. We perform CSAEC over 10 iterations on all datasets. It can be seen that on WIKI and NUS-WIDE, as the number of iterations increases, the loss value continues to decrease. After fewer iterations, the loss has been reduced and stabilized, and the method is considered to be convergent in the end.

## Discussion

The research on cross-modal retrieval technology has attracted much attention and is beginning to be put into practice. In addition, the semantic gap between the low-level features and high-level semantic features in the multi-modal dataset is a huge challenge. The bottleneck in accuracy and quality lies in the key factors. Researchers work on the construction of similarity constraints through category labels, but the methods are limited. Study the special correlation between multi-modal data is of great urgency.

Semantic information is significant knowledge retained during querying. Different forms of data have different feature spaces, but they have the same semantic space. Data with the same semantics are related in various forms. Semantic information can be used not only to indicate the degree of association between multiple modalities, but also to indicate the connections within each modality.

In this work, an effective cross-modal retrieval method CSAEC is proposed. By embedding mapping consensus on multi-modal data, while retaining the original feature information and semantic information, a semantic code vector is obtained. The paired encoder–decoders are linearly symmetric, returning feature projections to the original data, minimizing reconstruction errors. Parameters are introduced in the objective function with regularization sparse constraints. Experiments show that the autoencoder effectively completes the query task and improves the retrieval performance.

Cross-modal retrieval technology involves basic knowledge related to mathematics, and statistics to meet the needs of the application. Also, CSAEC can be applied to fields related to computers and networks such as deep and subspace learning. Further, CSAEC will play a great role in the field of recognition and analysis. In the next step, characteristics of the human body, such as facial expression and body movement, can be used on the deep neural network model to perform simultaneous features on multiple modal learning. Datasets can be unified to the same feature space as semantic expression through multiple nonlinear transformations. CSAEC can restore more similarities between image and text information for feature extraction. The model takes into account of different modalities and the importance of tasks for machine learning. The model breaks through the obstacles in traditional methods, using deep learning methods innovatively to convert multi modal data into abstract expression, which can get better accuracy and achieve better results in recognition.

## Methods

### Related work

Cross-modal similarity learning has aroused great attention in the academic community. However, the heterogeneity of data and the existence of semantic differences makes this problem challenging. At present, the two most common measurement methods are maximizing correlation and minimizing Euclidean distance^25^. The typical methods to maximize correlation are CCA^23^ and improved methods, learning a latent space that maximizes the correlation between the projection features of the two modalities. Reference^26^ used CCA to obtain the shared potential space of 2D and 3D facial images corresponding to people. PLS and BLM are methods to minimize Euclidean distance. Sharma and Jacobs^27^ used PLS to achieve heterogeneous facial recognition in different poses, high-resolution and low-resolution facial images, and between photos and sketches. Bilinear models (BLM) are used for cross-media retrieval and heterogeneous face recognition^2^.

**Figure 4:**
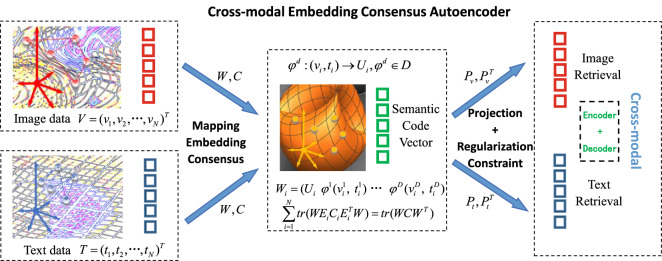
The process of CSAEC. We map the datasets to an embedding space, learn projections by multi-modal semantic autoencoder and reconstruct original features. $(V\;T)$ is the original data matrix, ${U_i}$ is a low-dimensional consensus vector of embedding consensus ${\varphi ^d}$, *W* is a low-dimensional embedding matrix, *C* is the corresponding semantic code. Two encoders ${P_v},{P_t}$ project image and text data into low-dimensional space *A*, and two decoders reproject *A* back to high-dimensional data.

An autoencoder is an unsupervised neural network model. It learns the hidden features of the input data, which is called encoding. Meanwhile, CSAEC reconstruct the original input data using the learned new features, which is called decoding. Autoencoders^28^ are trained models for learning potential representations of a set of data. CSAEC uses training data sets to copy the input information to the output. Therefore, the underlying representation is a valid attribute. Some scholars have proposed deformation methods for autoencoders. Reference^15^ correlated potential representations of two single-mode autoencoders. Kodirov et al.^16^ learned the semantic code vectors of latent space. Lange et al.^29^ combined the training of deep autoencoders (for learning compact feature spaces) with RL algorithms (for learning strategies). RL is short for Reinforcement Learning. Tara et al.^30^ used the training set to apply the AE-BN mode. The traditional autoencoder simply seeked potential representations to reconstruct the original data, and the method conducted the similarity with semantic code vectors. Inspired of related work, we improve existing methods and constructs a set of cross-modal semantic autoencoder with embedding consensus (CSAEC). The process is shown in Fig. 4. The paired image-text data is uniformly mapped to a low-dimensional embedding space, the manifold structure is retained, and the original information is converted into corresponding semantic code vectors. The consensus matrix and semantic code matrix are continuously updated. Further, by learning the image and text projection matrices, the encoders are used to associate them with corresponding semantic codes, and the decoder is reprojected back to the high-dimensional data. In addition, regularization and sparse constraints are performed on the decoder. Balanced parameters are used to reconstruct the original features. As a result, the method performs effectively on the retrieval of multi-modal information.

### Embedding consensus

Denote $(V\;T)$ the original data matrix, where $V = {({v_1},{v_2}, \ldots ,{v_N})^T}$ is the image information and $T = {({t_1},{t_2}, \ldots ,{t_N})^T}$ is the text information. The vector $({v_i},{t_i})$ represents the *i*-*th* row of information and $(v_i^d,t_i^d)$ represents the *d*-*th* dimension of the data $({v_i},{t_i})$. Mapping consensus mainly deals with the problem of multi-mapping disagreements. Since every data point is different, according to the mapping process, the representation of the same data point can be mapped into the latent embedding space. In this occasion, mapping conflict may occur. The reason is that the data point is unique which leads to different mapping results. The aim of mapping consensus is preserving validity of mappings and avoid mapping conflict. Considering of a fixed object $({v_i},{t_i})$ represented in different dimensions $(v_i^d,t_i^d)(d=1,2,\ldots ,D)$, we set ${\varphi ^d}:({v_i},{t_i}) \rightarrow {U_i}(i=1,2\ldots ,N)$ for each value of *d*, where ${U_i}$ is the definite representation of this point in latent embedding space and ${\varphi ^d}$ is the latent embedding mapping for *d*-th dimension. Embedding consensus matrix realizes the unity of each pair of image and text information mapping results, and further learns the semantic code vector. Manifold dimension reduction preserves the local geometry of the original data points. To prevent the results from being affected by the noise data, the parameter $\gamma _i^d$ is introduced. When sum up all of the *d* dimensions of $(v_i^d,t_i^d)(d=1,2,\ldots ,D)$, the $\sum \nolimits _{d = 1}^D {\gamma _i^d}$ can be transformed into $diag({\gamma _i})$. So we get

$$\begin{aligned} {\Gamma _{C(i)}}&= \sum \limits _{d = 1}^D {\gamma _i^d{{\left\| {{\varphi ^d} (v_i^d,t_i^d) - {U_i}} \right\| }^2}} \\&= diag({\gamma _i})tr\left( \left( \begin{array}{c} {\varphi ^1}(v_i^1, t_i^1) - {U_i}\\ \ldots \\ {\varphi ^D}(v_i^D,t_i^D) - {U_i} \end{array} \right) ({\varphi ^1}(v_i^1, t_i^1) - {U_i} \,,\;\ldots ,\;{\varphi ^D}(v_i^D,t_i^D) - {U_i})\right) \\&= tr\left( {W_i}\left( \begin{array}{l} - e_{D + 1}^T\\ {I_{D + 1}} \end{array} \right) diag({\gamma _i})({e_{D + 1}}\,{I_{D + 1}})W_i^T\right) \\&= tr({W_i}{C_i}W_i^T) \end{aligned}$$

${W_i} = ({U_i}\,{\varphi ^1}(v_i^1, t_i^1)\,,\;\ldots ,\;{\varphi ^D}(v_i^D, t_i^D))$ is a low-dimensional embedding matrix, which retains the manifold structure of the original information. Let

$$\begin{aligned} \varphi = diag({\varphi ^1}\,,\;\ldots ,\;{\varphi ^D}), {C_i} = \left( \begin{array}{l} - e_{D + 1}^T\\ {I_{D + 1}} \end{array} \right) diag({\gamma _i})({e_{D + 1}}\,{I_{D + 1}}) \end{aligned}$$

the data is transformed into the corresponding semantic code vector by embedding the consensus matrix. To eliminate the influence of noise, when the mapping result of data $({v_i},{t_i})$ is abnormal, $\gamma _i^d$ tends to 0. The corresponding features are extracted using the original image and text information. ${W_i}$ can be written as ${W_i} = W{E_i}$ , where ${E_i} = (e_i^T \,,\;\ldots ,\;e_{N + (i - 1)D + 1}^T\,,\;\ldots ,\;e_{N + iD}^T)$ is the feature matrix.

Sum the *N* components of images and text in each dimension

$$\begin{aligned} {\Gamma _C}&= \sum \limits _{i = 1}^N {{\Gamma _{C(i)}}} =\sum \limits _{i = 1}^N {tr({W_i}{C_i}W_i^T)} \\&= \sum \limits _{i = 1}^N {tr(W{E_i}{C_i}E_i^TW)} = tr(WC{W^T}) \end{aligned}$$

Denote

$$\begin{aligned} W= & {} (U\;{\varphi ^1}(v_1^1, t_1^1)\,,\;\ldots ,\;{\varphi ^D}(v_1^D, t_1^D) \,,\;\ldots ,\;{\varphi ^1}(v_N^1, t_N^1)\,,\;\ldots ,\;{\varphi ^D}(v_N^D, t_N^D)) \\ C= & {} \sum \limits _{i = 1}^N {{E_i}{C_i}E_i^T} = D - H \end{aligned}$$

where *H* is the correlation matrix between the mapping points and the original data points, and *D* is the diagonal matrix. Using matrix *C*, image and text information can be converted into corresponding semantic codes. Furthermore, let $\Phi = ({\varphi ^1}(v_1^1, t_1^1)\, \ldots {\varphi ^D}(v_1^D, t_1^D)\, \ldots {\varphi ^1}(v_N^1, t_N^1)\, \ldots {\varphi ^D}(v_N^D, t_N^D))$, the final expression is

$$\begin{aligned} {\Gamma _C}&= tr(WC{W^T})\\&= tr((U\;\Phi )\left( {\begin{array}{cc} {{C_{11}}}&{}{{C_{12}}}\\ {{C_{21}}}&{}{{C_{22}}} \end{array}} \right) \left( \begin{array}{c} {U^T}\\ {\Phi ^T} \end{array} \right) )\\&= tr(U{C_{11}}{U^T}) + tr(\Phi {C_{21}}{U^T}) + tr(U{C_{12}}{\Phi ^T}) +tr(\Phi {C_{22}}{\Phi ^T}) \end{aligned}$$

The variables in the objective function are relatively complex, and each univariate is solved by using an iterative update method.

$$\begin{aligned} \mathop {\text{min} }\limits _{\Phi ,C,U} {\Gamma _C}&= tr(WC{W^T})\\&= tr(U{C_{11}}{U^T}) + tr(\Phi {C_{21}}{U^T}) + tr(U{C_{12}}{\Phi ^T}) +tr(\Phi {C_{22}}{\Phi ^T}) \end{aligned}$$

First, fix *C*, *U* and update $\Phi .$

Since $\Phi = (V\;T){\varphi ^T}$, the objective function can be transformed into a solution for a single variable

$$\begin{aligned} \Gamma (\Phi )&= \left\| {\Phi - {U^T}} \right\| _F^2\\&= \left\| {(V\;T){\varphi ^T} - {U^T}} \right\| _F^2 \end{aligned}$$

where

$$\begin{aligned} (V\;T)= \,& {} ((v_1^1, t_1^1)\,,\;\ldots ,\;(v_1^D, t_1^D) \,,\;\ldots ,\;(v_N^1, t_N^1)\,,\;\ldots ,\;(v_N^D, t_N^D)) \\ {\varphi ^T}=\, & {} diag(\varphi \,,\;\ldots ,\;\varphi ) \\ {U^T}=\, & {} ({U_1}\,,\;\ldots ,\;{U_1}\,,\;\ldots ,\;{U_N}\,,\;\ldots ,\;{U_N}) \\ \end{aligned}$$

Find the partial derivatives of ${\varphi ^T}$

$$\begin{aligned} \frac{{\partial \Gamma }}{{\partial {\varphi ^T}}}=\, & {} 2((V\;T){\varphi ^T} - {U^T})(V\;T) = 0 \\ {\varphi ^T}= \,& {} {(V\;T)^{ - 1}}{U^T} \\ \end{aligned}$$

Second, fix $\Phi ,U$ and update *C*.

The expression becomes

$$\begin{aligned} \Gamma (C) = tr(WC{W^T}) \end{aligned}$$

The solution of *C* can be referenced to^31^.

Third, fix $\Phi ,C$ and update *U*

The update process is transformed into a single variable *U*

$$\begin{aligned} \Gamma (U) = tr(U{C_{11}}{U^T}) + tr(\Phi {C_{21}}{U^T}) + tr(U{C_{12}}{\Phi ^T}) \end{aligned}$$

Find the partial derivative of *U*

$$\begin{aligned}&2{C_{11}}{U^T} + \Phi {C_{21}} + {\Phi ^T}{C_{12}}^T = 0 \\&U = {( - {(2{C_{11}})^{ - 1}}(\Phi {C_{21}} + {\Phi ^T}{C_{12}}^T))^T} \end{aligned}$$

### Cross-modal semantic autoencoder

By mapping the image and text to the embedding consensus space, CSAEC can contain enough raw data information. $V \in {R^{d_v \times n}},T \in {R^{d_t \times n}}$ denote the visual and textual feature matrices, respectively, where $d_v$ and $d_t$ are the visual and textual feature dimensionalities. The following is to learn the projection matrix $P_v \in {R^{d \times d_v}},P_t \in {R^{d \times d_t}}$ separately: the encoder connects the image and text projection with the semantic code vector *C*, and the decoder is restricted so that the code vector can reconstruct the original features of the image and text. The encoder and decoder are linearly symmetric. Two encoders ${P_v},{P_t}$ project image and text data into low-dimensional space *A*, and two decoders reproject *A* back to high-dimensional data. The hidden layer contains both image and text information.

For the image data, the embedding form of the automatic encoder is used to represent the information of the original features. The image-text paired representation should be unified, since in the retrieval stage, when the query information is given, the query will be sorted according to the similarity. So, we get

$$\begin{aligned} {P_v}V = A \end{aligned}$$

where $A \in {R^{d \times n}}$ represents *n* groups of training texts in a *d*-dimensional hidden space. The additional reconstruction task imposes a new constraint in learning of the projection function so that the projection must preserve all the information contained in the original textual features. For image modality, we also adopt an autoencoder to let the embeddings contain information from original visual features. We hope the representations of image-text pairs in the hidden space to be uniform. This form is a binding linear autoencoder^18^ and has only one hidden layer.

For text data, to make sure of the low-dimensional ability to restore the original information points, let

$$\begin{aligned} {P_t}T = A \end{aligned}$$

For each data point $v_i$($\hbox {i} = 1$, 2, …, *N*), it can be approximated as a linear combination of all the other samples. Based on the mapping consensus we have proposed, the datasets ${\varphi ^d}:({v_i},{t_i}) \rightarrow {U_i}(i=1,2\ldots ,N)$, we set $d=d_v=d_t$. In this way, the feature matrices $V \in {R^{d \times n}},T \in {R^{d \times n}}$, $P_v \in {R^{d \times d}},P_t \in {R^{d \times d}}$. Then by imposing sparsity on the matrix *A* and the projection matrix ${P_v}$ to the process of reconstruction, the optimal sparse combination matrix *A* and projection matrix ${P_v}$ can be obtained by solving the problem

$$\begin{aligned} \mathop {\text{min} }\limits _{{P_v},A} \sum \limits _{i = 1}^N \left( \left\| {{P_v}^Tv_i - {P_v}^TVa_i} \right\| _F^2 + {\left\| A \right\| _1}\right) \end{aligned}$$

where $a_i$ is the *i*th column vector of the matrix *A*. As in the manifold learning methods, $P_vV$ should satisfy the orthogonal constraint. Through the sparsity constraint, the information captured by *A* can be used to search the relevant features and eliminate the effect of noise features. The function for structure learning is formulated as

$$\begin{aligned} \mathop {\text{min} }\limits _{{P_v},A} \left( \left\| {{P_v}^TV - {P_v}^TVA} \right\| _F^2 + {\left\| A \right\| _1}\right) \end{aligned}$$

According to the expressions above, a multi-modal autoencoder can be obtained. Also, we make sure that the hidden layer contains enough semantic information. The hidden representation of the data is associated with the semantic code vector *C*. Considering the similarity between different modalities, we use tag information to standardize the potential representation of the autoencoder. The reference^18^ has minimized the function by summing up the low-dimensional information of visual and text datasets. This method^18^ relaxed the constraints and rewrite the objective of multi-modal autoencoder. In this way, the results have been improved. In retrieval phase, when a query is given, documents are sorted according to their similarity to the query. To guarantee the projected images and texts containing both semantic information and original feature information, we propose an improved autoencoder. On this basis, a regularization sparse constraint on the low-dimensional matrix *A* is added to obtain the final objective function

$$\begin{aligned}{} &\mathop {\rm{min} }\limits _{{P_v},{P_t},A} \left\| {{P_v}V +{P_t}T - A} \right\| _F^2 + \beta \left(\left\| {{P_v}^TV - {P_v}^TVA} \right\| _F^2 + \left\| {{P_t}^TT - {P_t}^TTA} \right\| _F^2\right)\\&\begin{array}{ll} &{} + \eta \left\| {A - C} \right\| _F^2 + {\left\| A \right\| _1} \\ s.t. &{} {P_v}^T{P_v} = I,P_v^TV{L_A}{V^T}{P_v} = I,\\ &{} {P_t}^T{P_t} = I,P_t^TT{L_A}{T^T}{P_t} = I,\\ &{} {A_{ii}} = 0 \end{array} \end{aligned}$$

where $\beta$ is the weight parameter for balancing the two types of data information, and is a parameter that determines importance of semantic code vector.

We also use alternating iterative updating methods to solve the objective function separately.

First, fix *A* and update ${P_v},{P_t}$

The solutions of the projection matrix ${P_v},{P_t}$ are similar. Let

$$\begin{aligned} {L_A} = (I - A){(I - A)^T}\ \end{aligned}$$

The expression is transformed into univariate functions of ${P_v}$

1 $$\begin{aligned}&\mathop {\text{min} }\limits _{{P_v}} \left\| {{P_v}V - A} \right\| _F^2 +\beta tr(P_v^TV{L_A}{V^T}{P_v}) \\&s.t. \quad {P_v}^T{P_v} = I,P_v^TV{L_A}{V^T}{P_v} = I, {A_{ii}} = 0 \end{aligned}$$

By finding partial derivatives of ${P_v}$, the specific solution method can be found in the reference^31^. Similarly, the update function of ${P_t}$ is

2 $$\begin{aligned}&\mathop {\text{min} }\limits _{{P_t}} \left\| {{P_t}T - A} \right\| _F^2 +\beta tr({P_t}^TT{L_A}{T^T}{P_t}) \\&s.t. \quad {P_t}^T{P_t} = I,{P_t}^TT{L_A}{T^T}{P_t} = I, {A_{ii}} = 0 \end{aligned}$$

Second, fix ${P_v},{P_t}$ and update *A* Find partial derivative of variable *A*

3 $$\begin{aligned}&(2A - {P_v}V - {P_t}T) \\&\quad + 2\beta ({V^T}{P_v}P_v^TV + {V^T}{P_v}P_v^TVA + {T^T}{P_t}P_t^TT + {T^T}{P_t}P_t^TTA) \\&\quad + \eta (A - C) + \frac{A}{{||A|{|_1}}} = 0 \end{aligned}$$

According to the solution method of LASSO problem^32^, matrix *A* can be updated.

Synthesizing the process above, we propose the cross-modal semantic autoencoder with embedding consensus.

**Table:** 

**Cross-Modal Semantic Autoencoder with Embedding Consensus (CSAEC)**
**Input:** Data matrix *V*, *T* and code vector *C*, parameters $\beta ,\eta$
**Output:** Projection matrices ${P_v},{P_t}$
**Initialize:** Use matrix *C* to initialize matrix *A*;
**Repeat:**
Fix *A*, and update ${P_v},{P_t}$ according to Eq.(1) and Eq.(2);
Fix ${P_v},{P_t}$, and update *A* according to Eq.(3);
**Until convergence.**
